# Radioenhancement with the Combination of Docetaxel and Ultrasound Microbubbles: In Vivo Prostate Cancer

**DOI:** 10.3390/pharmaceutics15051468

**Published:** 2023-05-11

**Authors:** Firas Almasri, Emmanuel H. Sakarya, Raffi Karshafian

**Affiliations:** 1 Department of Physics, Toronto Metropolitan University, Toronto, ON M5B 2K3, Canada; 2 Biomedical Engineering Department, International University of Science and Technology in Kuwait, Ardiya 92400, Kuwait; 3 Institute for Biomedical Engineering, Science and Technology (iBEST), A Partnership Between Toronto Metropolitan University and St. Michael’s Hospital, Toronto, ON M5B 1T8, Canada; 4 Keenan Research Centre for Biomedical Science, St. Michael’s Hospital, Toronto, ON M5G 0A3, Canada

**Keywords:** radiotherapy, radioenhancement, chemotherapy, ultrasound, and microbubble therapy

## Abstract

Using an in vitro prostate cancer model, we previously demonstrated the significant enhancement of radiotherapy (XRT) with the combined treatment of docetaxel (Taxotere; TXT) and ultrasound-microbubbles (USMB). Here, we extend these findings to an in vivo cancer model. Severe combined immune-deficient male mice were xenografted with the PC-3 prostate cancer cell line in the hind leg and treated with USMB, TXT, radiotherapy (XRT), and their combinations. The tumors were imaged with ultrasound pre-treatment and 24 h post-treatment, following which they were extracted for the histological analysis of the tumor-cell death (D_N_; H&E) and apoptosis (D_A_; TUNEL). The tumors’ growths were assessed for up to ~6 weeks and analysed using the exponential Malthusian tumor-growth model. The tumors’ doubling time (V_T_) was characterized as growth (positive) or shrinkage (negative). The cellular death and apoptosis increased ~5-fold with the TXT + USMB + XRT (D_n_ = 83% and D_a_ = 71%) compared to the XRT alone (D_n_ = 16% and D_a_ = 14%), and by ~2–3-fold with the TXT + XRT (D_n_ = 50% and D_a_ = 38%) and USMB + XRT (D_n_ = 45% and D_a_ = 27%) compared to the XRT. The USMB enhanced the cellular bioeffects of the TXT by ~2–5-fold with the TXT + USMB (D_n_ = 42% and D_a_ = 50%), compared with the TXT alone (D_n_ = 19% and D_a_ = 9%). The USMB alone caused cell death (D_n_ = 17% and D_a_ = 10%) compared to the untreated control (D_n_ = 0.4% and D_a_ = 0%). The histological cellular bioeffects were correlated with the changes in the ultrasound RF mid-band-fit data, which were associated with the cellular morphology. The linear regression analysis displayed a positive linear correlation between the mid-band fit and the overall cell death (R^2^ = 0.9164), as well as a positive linear correlation between the mid-band fit and the apoptosis (R^2^ = 0.8530). These results demonstrate a correlation between the histological and spectral measurements of the tissue microstructure and that cellular morphological changes can be detected by ultrasound scattering analysis. In addition, the tumor volumes from the triple-combination treatment were significantly smaller than those from the control, XRT, USMB + XRT, and TXT + XRT, from day 2 onward. The TXT + USMB + XRT-treated tumors shrank from day 2 and at each subsequent time-point measured (V_T_ ~−6 days). The growth of the XRT-treated tumors was inhibited during the first 16 days, following which the tumors grew (V_T_ ~9 days). The TXT + XRT and USMB + XRT groups displayed an initial decrease in tumor size (day 1–14; TXT + XRT V_T_ ~−12 days; USMB + XRT V_T_ ~−33 days), followed by a growth phase (day 15–37; TXT + XRT V_T_ ~11 days; USMB + XRT V_T_ ~22 days). The triple-combination therapy induced tumor shrinkage to a greater extent than any of the other treatments. This study demonstrates the in vivo radioenhancement potential of chemotherapy combined with therapeutic ultrasound-microbubble treatment in inducing cell death and apoptosis, as well as long-term tumor shrinkage.

## 1. Introduction

Radiotherapy (XRT) is a fundamental modality in cancer therapy and has achieved significant clinical success. The use of XRT requires large doses (>40 Gy), which are generally delivered in small, tolerable fractions (~2 Gy) [1] and act predominantly by causing single- and double-stranded breaks in the cellular DNA of cancerous tissues and their supporting vasculature [2]. The perturbation of the microvascular networks of tumors has been shown to enhance the effects of radiotherapy on cancer cells. For example, radiation-induced endothelial-cell apoptosis results in the secondary cell death of MCA/129 fibrosarcoma and B16F10 melanoma [3]. However, achieving microvascular damage requires high radiation doses (a minimum of 8–10 Gy per single dose), which limits its applicability [4]. In addition, depending on tumor type and characteristics, localizing radiation damage to tumor tissue remains a challenge, which limits the radiation dose administered [5]. Therefore, different strategies to enhance and maximize the effect of radiation are under investigation, such as strategies targeting the DNA damage and repair mechanisms induced by XRT [6,7,8], as well as strategies that target tumors’ vascular supply [9,10]. These include pharmaceutical agents, such as chemotherapy and antivascular drugs [11,12,13], nanoparticles, such as gold [14,15,16,17], hyperthermia [18,19,20,21] and ultrasound-microbubbles (USMB) [22,23].

The application of USMB with either radiotherapy or chemotherapy has been shown to improve the efficacy of the latter therapies in various preclinical studies (reviewed in [24]), with the potential to reduce radiotherapy and chemotherapy doses to limit associated side effects on healthy tissues. The use of USMB has been shown to be a promising strategy to enhance cancer therapies due to its ability to disrupt a tumor’s vascular network [24]. Microbubbles are micron-sized gaseous spheres encapsulated by a stabilizing material of lipids or proteins. Several commercially sold microbubble products are used as contrast agents for the diagnostic imaging of tissues. More recently, microbubbles were re-purposed for their therapeutic effects [25]. Their small size (with a median of 3 μm) allows them to circulate throughout the vascular system following peripheral venous injection [26] and to undergo cavitation within the ultrasound field, producing a localized bioeffect on the target tissue. The combination of USMB with radiotherapy or chemotherapy has shown a significant therapeutic improvement in preclinical studies [22,27,28,29,30,31,32].

The application of USMB alone has been shown to synergistically enhance the effects of radiotherapy in a variety of tumor models, and it was demonstrated that low-dose radiotherapy combined with USMB can achieve similar therapeutic effects to high-dose radiotherapy [22,29,33,34]. The radioenhancement effects of USMB have been demonstrated in preclinical studies on breast [34,35], bladder [29], and colorectal carcinoma [36], fibrosarcoma [28,35], melanoma [37], and prostate cancers [22,27,35]. Czarnota et al. demonstrated that a single 2-Gy radiation dose combined with USMB increased murine-prostate-tumor-cell death (2–3-fold, compared to radiation-only treatment groups) and prolonged the survival of PC-3 prostate-carcinoma-xenografted mice. The radioenhancement achieved with USMB was associated with the endothelial-cell disruption of the tumor microvasculature [22]. Mechanistic studies revealed that USMB induces apoptosis, in part, by increasing sphingomyelinase activity on the cell surface, which enhances the production of ceramide, a mediator of cell death [23,33].

The application of USMB can enhance chemotherapy’s efficacy by potentially increasing the local concentration of chemo-drugs in the tumor environment associated with vascular leakage and damage [38,39,40]. Chemotherapy is a standard cancer treatment targeting dividing cells, both healthy and malignant. As a result, the therapeutic application of chemotherapy is limited by the detrimental side effects resulting from the death of healthy cells, such as epithelial and hematopoietic cells [11,41]. Moreover, the efficacy of chemotherapeutic agents depends on the concentration of the drug in the tumor environment [42]. Consequently, studies have leveraged USMB to enhance the local concentrations of chemotherapeutics by conjugating drug molecules to microbubbles (MBs) and/or by increasing the permeability of blood vessels and cell membranes [26,43,44,45,46]. Goertz et al. demonstrated enhancement of the anti-tumor activity of docetaxel when combined with USMB [40]. Indeed, it is well known that the efficacy of the taxane drug, docetaxel, is increased when combined with vascular disrupting agents. In clinical settings, docetaxel is used in conjunction with radiotherapy to treat solid tumors [47]. There is an extensive body of research (both clinical and scientific) demonstrating the synergistic effects of combining docetaxel and radiotherapy [48,49].

There is ample evidence that the therapeutic effects of radiotherapy are enhanced with both chemotherapy and USMB. Furthermore, evidence suggests a three-way interaction between the treatment options, which warrants an investigation of the use of the three treatments as a combination therapy. This strategy may improve the therapeutic targeting of both the vascular and malignant compartments of a tumor. We have shown that the combination of the taxane, docetaxel (Taxotere, TXT), USMB, and XRT produced a synergistic enhancement of prostate-cancer-cell death in vitro [50]. We treated in vitro-suspended PC-3 prostate carcinoma cells with different combinations of XRT, TXT, and USMB. The triple-combination therapy achieved significantly greater levels of cell death (~2% viability) than the double-combination treatments (~20–60% viability) and the single treatments (~55–75% viability). Moreover, the levels of cell death increased with the microbubble concentration and chemotherapy-drug concentration. To the best of our knowledge, no other studies have investigated the use of triple-combination therapy for treating cancer based on USMB, chemotherapy, and radiotherapy. Instead, studies have investigated the efficacy of double-combination therapies consisting of chemotherapy + USMB [32,40,51], radiotherapy + USMB [22,29,33,34], and chemotherapy + radiation [48,49]. Here, we expand on our in vitro findings by investigating the radioenhancement potential of TXT + USMB in an in vivo setting. Mice xenografted with the PC-3 prostate carcinoma cell line were treated with various combinations of TXT, USMB, and XRT, and assessed for radioenhancement at both short-term (24 h post-treatment) and long-term (~5–6 weeks post-treatment) follow-up intervals. Our results indicate the enhancement of radiotherapy and complete tumor ablation when pre-treating mice with TXT + USMB. These findings indicate that the triple-combination therapy may be a superior treatment option (compared to single and double-combination therapies) for prostate carcinoma and/or other solid tumors. Additionally, the increased efficacy of the triple-combination therapy may allow clinicians to reduce toxic radiation and chemotherapy doses while maintaining significant tumor responses.

## 2. Materials and Methods

### 2.1. In Vivo Tumor Model

Severe combined immunodeficient (SCID) male mice (Charles River Laboratory International Inc., Canada) ~6 weeks of age and weighing ~25 g were injected subcutaneously with 50 μL (10^6^ cells) of suspended prostate cancer cells in their right hind legs, as previously described [22,33]. The human prostate cancer cell line (PC-3, American Type Culture Collection, Manassas, VA, USA) was cultured in RPMI-1640 growth medium (Wisent, St Bruno, QC, Canada) supplemented with 1% penicillin/streptomycin (Gibco, Life Technologies, Burlington, ON, Canada) and 10% fetal bovine serum (Thermo Scientific Hyclone, Logan, UT, USA). The tumor cells were collected for injection using 0.05% Trypsin-EDTA (Gibco, Life Technologies, Burlington, ON, Canada). Within 4–6 weeks, tumors reached sizes of ~6–7 mm in diameter, following which they were treated with varying combinations of chemotherapy (Taxotere; TXT), USMB, and radiotherapy (XRT). Animals were sacrificed 24 h after treatment. A total of 8 different treatment conditions were investigated, with four mice per condition (32 animals in total). The conditions were as follows: (1) untreated control, (2) TXT, (3) USMB, (4) TXT + USMB, (5) XRT, (6) TXT + XRT, (7) USMB + XRT, and (8) TXT + USMB + XRT. In addition, a second cohort of mice (four mice per condition and five treatment groups; 20 animals in total) was used to conduct a tumor-growth-delay study. The treatment groups were as follows: (1) untreated control, (2) XRT, (3) TXT + XRT, (4) USMB + XRT, and (5) TXT + USMB + XRT. Prior to imaging and treatments, animals were anesthetized by intraperitoneal injection of 0.1 mL of solution comprising ketamine (100 mg/kg), xylazine (5 mg/kg), and acepromazine (1 mg/kg). Animals were sacrificed while anesthetized using cervical dislocation. All animals were handled in accordance with institutional animal care policies.

### 2.2. Chemotherapy: Docetaxel (TXT)

Docetaxel (Taxotere, TXT) was obtained through Sunnybrook Health Sciences Center from Aventis Pharmaceuticals (Bridgewater, NJ, USA). Following a previously published protocol [40], TXT was diluted in an ethanol–saline solution at a concentration of 1 mg/mL and administered at a dose of 5 mg/kg through a tailvein catheter, and then flushed with 100 μL saline.

### 2.3. Ultrasound Microbubble (USMB) Therapy

Tumor xenografts were treated with USMB following descriptions in previously published studies [22,27,33]. The microbubbles employed were commercially available Definity^TM^ microbubbles (perfluoropropane gas/liposome shell; Lantheus Medical Imaging, Inc., North Billerica, MA, USA). Definity^TM^ microbubbles are composed of octafluoropropane gas (C_3_F_8_) encapsulated in a lipid bilayer of 1,2-dipalmitoyl-sn-glycero-3-phosphocholine (DPPC), 1,2-dipalmitoyl-sn-glycero-3-phosphoethanolamine (DPPE) and 1,2-dipalmitoyl-sn-glycero-3-phosphate (DPPA). The Definity^TM^ microbubbles were administered at a concentration of 2 μL/g through a tail-vein catheter, followed by a 100-μL saline flush; this corresponded to 50 μL (6.0 × 10^8^ microbubbles) of undiluted Definity^TM^ for a 25 g animal. Ultrasound treatment of mice was conducted in a water tank (35 °C), wherein mice were positioned vertically for acoustic coupling with the ultrasound transducer. The ultrasound transducer (IL0509HP, Valpey Fisher Inc., Hopkinton, MA, USA) was focused on the tumor, and controlled pulses were generated and amplified by a waveform generator (AWG520, Tektronix Inc, Beaverton, OR) and power amplifier (RPR4000, Ritec Inc, Warwick, RI, USA), respectively. The transducer contained a 28.6-mm element diameter focused at 85 mm, with a focused central frequency of 500 kHz, and produced a −6-dB ultrasound beam (31-mm beam width). The transducer was positioned approximately normal to the surface of the tumor in the hind leg and covered the whole tumor area. The ultrasound sequence (2 min in duration) comprised a 50-ms sequence with a 10-s sequence-repetition period, and each sequence comprised a 32-μs pulse duration (16-cycle tone burst) and 3-kHz pulse-repetition frequency. Mice were administered TXT and/or MBs with an ultrasound sequence consisting of 350 kPa of peak negative pressure followed by a second MB injection (5 min following the first ultrasound sequence) with an ultrasound sequence corresponding to 580 kPa of peak negative pressure.

### 2.4. Radiotherapy (XRT)

Mice were irradiated immediately following chemotherapy and/or USMB treatments, as previously described [22]. A 3-mm-thick lead sheet covered the whole of each mouse’s body while leaving the tumor area exposed to the X-ray irradiator (Faxitron Xray Corporation, Lincolnshire, IL, USA). Tumors were exposed to an 8-Gy single-fraction dose with a 160-kVp and 200-cGy/min dose rate at a source-to-surface (SSD) distance of 35 cm.

### 2.5. Spectral Analysis of Ultrasound Radiofrequency Data

Ultrasound radiofrequency (RF) was acquired using a high-frequency ultrasound imaging system (VEVO 770, VisualSonics, Toronto, ON, Canada) with a single-element RMV (real-time micro visualization) transducer. The RF data were acquired pre-treatment and 24 h post-treatment, as described previously [22,33]. The scan began from the upper leg of the mouse and moved toward the foot. The RF data were acquired by collecting 250 RF lines per image. The region of interest was rectangular in shape and equivalent in all samples. The RF data were analyzed by calculating the difference in decibels (dBs) pre- and post-treatment to obtain the mid-band fit parameter. Larger mid-band fit values resulted from RF backscattering caused by an increase in apoptotic cells in the tumor tissues. The mid-band fit values were calculated based on an 18–30-MHz range.

### 2.6. Tissue Histology: H&E and Tunel

Tumor samples were excised 24 h post-treatment, fixed overnight in 1.0% paraformaldehyde, and then embedded in paraffin blocks. Tumor samples were sectioned from distal to proximal ends of the tumor. Slices were cytospinned at 2000× *g* and fixed for 30 min, followed by hematoxylin and eosin (H&E) and terminal deoxynucleotidyl transferase dUTP nick-end labeling (TUNEL) staining. Hematoxylin and eosin (H&E) staining was used to visualize tumor tissue and cellular morphology by differentially staining various intracellular components and the extracellular space [52,53]. The use of H&E staining was applied previously to identify and quantify necrotic areas of tumors [54,55], including prostate cancer [56]. Necrotic zones correspond to anuclear and acellular regions identified by a faint pink appearance resulting from diffuse eosin staining [54]. Tumor images were captured using a Leica DC100 microscope coupled to a Leica DC100 video camera wired to a 2-GHz PC running Leica IM1000 software (Leica GmbH). Analysis of necrotic areas was performed as previously described [54,55,56,57]. Necrotic regions were traced using selection tools available on ImageJ, and necrosis was presented as a percentage of the whole tumor area. The experimenter was blinded to the treatment groups during quantification. Terminal deoxynucleotidyl transferase dUTP nick-end labeling (TUNEL) is a common staining technique used to detect apoptotic cells [58,59]. Tumor sections were stained using an in-lab TUNEL kit provided by the University Health Network (UHN), which works similarly to EMD Millipore’s Apoptag Kit. Apoptotic cells were detected by labeling the 3′hydroxyl termini of fragmented DNA with a modified nucleotide and visualized immunohistochemically with a peroxidase reporter molecule. Stained sections were visualized using bright-field microscopy, and images were captured using a Leica DC100 microscope coupled to a Leica DC100 video camera wired to a 2-GHz PC running Leica IM1000 software (Leica GmbH). Immunostained areas were traced using ImageJ selection tools and reported as a percentage of the whole tumor area (% apoptosis). The experimenter was blinded to the treatment groups during quantification.

### 2.7. Growth Delay and Survival

Tumors were measured with a vernier caliper three times per week throughout the duration of the study. The tumor volume was determined using the following formula, which accounts for its elliptical shape [60]:

$$V_{Tumour}\left[{\mathrm{mm}}^{3}\right]=\frac{\pi }{6}l\cdot w\cdot h$$

where *l*, *w*, and *h* are the tumor length, width, and height in millimeters, respectively. Tumor-volume measurements began on treatment day (day 1) and up to 41 days following treatment day, until mice were euthanized due to ethical endpoints associated with tumor burden. The change in the tumor volume was characterized based on the exponential (exponential (Malthusian) growth model, nonlinear least-square fit, v9.5.0 Graphpad Software Inc. (San Diego, CA, USA) [61]:

$$V_{Tumour}=V_{0}\cdot e^{k\cdot t}$$

where *V*_0_ is the initial volume, *k* is the rate constant (1/day), and *t* is the time in days. Volumetric data for the treatment conditions TXT + XRT and USMB + XRT were analyzed by separating curves into shrinkage and growth phases and fitting both curve phases to the exponential model. The results are reported in terms of tumor-doubling time (shrinkage and growth). The tumor-doubling time (V_T_), calculated using ln(2)/*k*, characterizes tumor growth (positive values) or shrinkage (negative values).

### 2.8. Statistical Analysis

Quantification of cell death (H&E) and apoptosis (TUNEL) stained sections and quantification of RF mid-band fit are reported as mean ± standard deviation. The differences between the treatments were analyzed by two-way ANOVA and with Tukey–Kramer multiple-comparisons test (α = 0.05). All analyses were performed using GraphPad Prism (Ver. 9.5.0, GraphPad Software Inc.).

## 3. Results

### 3.1. Cell Death and Apoptosis

**Figure 1:**
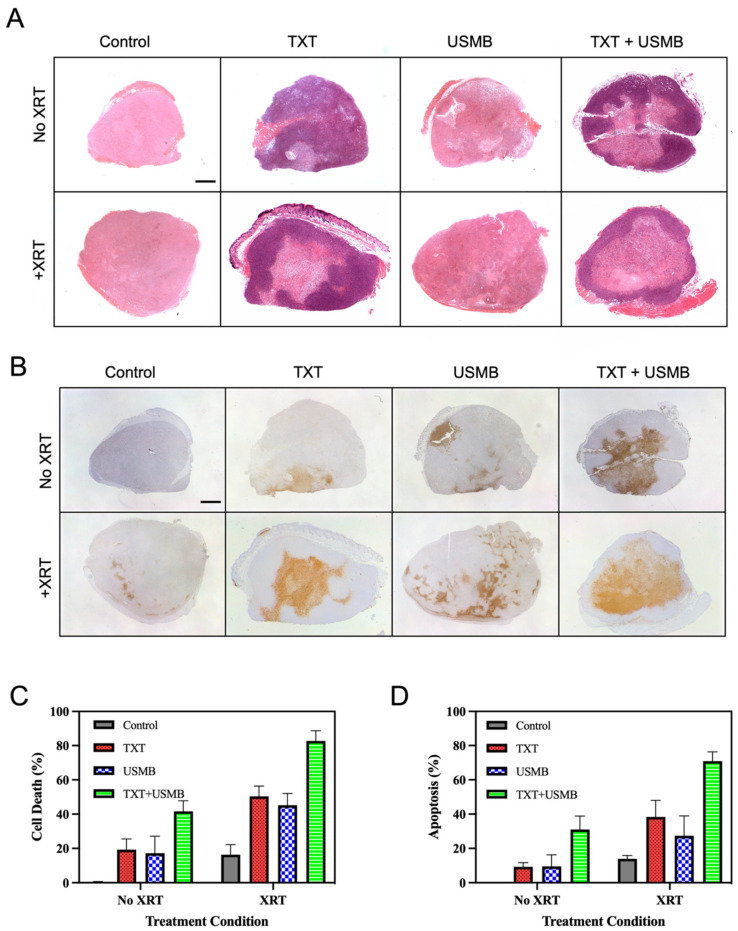
Cell-death assessment on PC-3-xenografted tumors treated with varying combinations of ultrasound-microbubbles (USMB), docetaxel (TXT), and radiotherapy (XRT). (**A**) Representative images of hematoxylin and eosin (H&E)-stained tumor sections and (**B**) representative images of TUNEL-stained tumor sections for eight different treatment groups. Cell death, corresponding to clear necrotic zones (D_n_) for H&E, is quantified in panel (**C**) and is presented as a percentage of the whole tumor area. The extent of apoptosis in each tumor, corresponding to TUNEL-positive staining (D_a_), is quantified in panel (**D**) and is represented as a percentage of the whole tumor area. Four animals were used per condition, with the following conditions: untreated control, TXT, USMB, TXT + USMB, XRT, TXT + XRT, USMB + XRT, and TXT + USMB + XRT. Values are expressed as mean ± SD. Statistical analysis was performed using a two-way ANOVA and Tukey’s multiple-comparison test. Differences between treatment means were found to be significant at an alpha level of 0.05. Scale bar 1 mm.

To investigate the effects of the combination therapies on the overall tumor-cell death and apoptosis, hematoxylin and eosin (H&E) and terminal deoxynucleotidyl transferase dUTP nick-end labeling (TUNEL) were employed, respectively. The tumors were extracted 24 h post-treatment, and the tumor sections were stained with H&E (Figure 1A) and TUNEL (Figure 1B). As expected, the single treatments resulted in similar levels of necrotic (D_n_) and apoptotic (D_a_) cell death, corresponding to 10–20% of the whole tumor area (Figure 1C,D). Compared to the untreated control (D_n_ = 0.379 ± 0.308%), the cell death was significantly increased with the single treatments of TXT (D_n_ = 19.255 ± 6.302%, *p* = 0.0072), USMB (D_n_ = 17.170 ± 9.984%, *p* = 0.0211), and XRT (D_n_ = 16.393 ± 5.824%, *p* = 0.0311), as indicated by the H&E. Compared to the untreated control (D_a_ = 0.000 ± 0.000%), the proportion of apoptosis in the tumors also increased (although this increase was not statistically significant) with the single treatments of TXT (D_a_ = 9.290 ± 2.389%, *p* = 0.5513), USMB (D_a_ = 9.513 ± 6.799%, *p* = 0.5229), and XRT (D_a_ = 13.953 ± 1.913%, *p* = 0.1207). Compared to the single treatments, the double-combination therapies produced greater levels of tumor-cell death (corresponding to 40–50% of the whole tumor area, Figure 1C,D; *p* < 0.05) and apoptosis (corresponding to 25–40% of the entire tumor area, Figure 1C,D; *p* < 0.05, with the exception of the XRT vs. USMB + XRT comparison). There were no statistically significant differences between the double-combination therapies (refer to Table 1 and Table 2 for *p*-values). Finally, the triple-combination therapy (D_n_ = TXT + USMB + XRT; 82.741 ± 5.968%) resulted in significantly greater levels of cell death than the TXT + USMB (D_n_ = 41.617 ± 6.202%, *p* < 0.0001), TXT + XRT (D_n_ = 50.346 ± 6.012%, *p* < 0.0001), and USMB + XRT (D_n_ = 45.244 ± 6.788%, *p* < 0.0001). We also observed significantly greater levels of apoptosis with the triple-combination therapy (D_a_ = 70.843 ± 5.418%) compared to the TXT + USMB (D_a_ = 31.045 ± 7.829%, *p* < 0.0001), TXT + XRT (D_a_ = 38.335 ± 9.678%, *p* < 0.0001), and USMB + XRT (D_a_ = 27.335 ± 11.604%, *p* < 0.0001). These results indicate an enhancement of radiotherapy in vivo when combining XRT with TXT + USMB.

### 3.2. Mid-Band Fit and Tissue Microstructure

**Figure 2:**
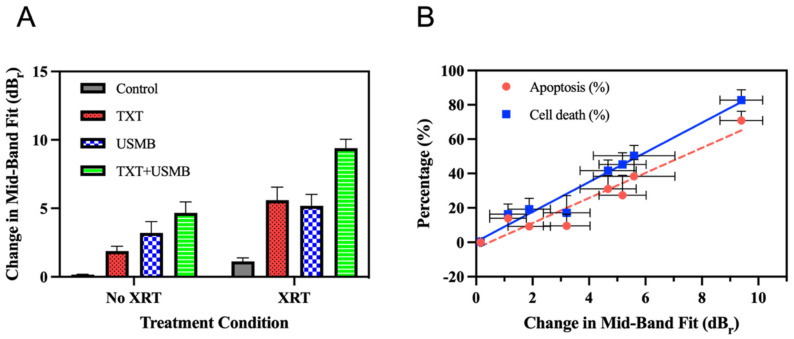
Changes in mid-band fit and correlation of mid-band-fit changes with histological assessment of tumor-cell death. (**A**) Bar graph depicting the differences between the mid-band-fit-ultrasound-parameter pre-treatment and 24-h post-treatment based on the amount of backscattered radiofrequency captured by high-frequency ultrasound imaging. Four animals were used for each of the following conditions: untreated control, TXT, USMB, TXT + USMB, XRT, TXT + XRT, USMB + XRT, and TXT + USMB + XRT. (**B**) The relationship between mid-band-fit change and percentage of apoptosis (red bullets and trendline) and mid-band-fit change and percentage of cell death (blue bullets and trendline) for all treatment conditions. Regression analysis comparing mid-band-fit changes with percentage of apoptosis generated a best-fit line with the following equation and coefficient of determination: y = 7.291x − 3.413 R^2^ = 0.8830. Regression analysis comparing mid-band-fit changes with percentage of cell death generated a best-fit line with the following equation and coefficient of determination: y = 8.640x + 0.4203 R^2^ = 0.9164. All values are expressed as mean ± SD. Differences between the treatment means were found to be significant at an alpha level of 0.05.

In addition to the histological assessment of the cell death, high-frequency ultrasound imaging was performed to provide information on the cellular morphologies of tumors. Radiofrequency (RF) measurements were acquired prior to and 24 h following the treatments. The changes observed in the radiofrequency measurements (calculated as changes in the mid-band fit) reflected the ultrasound scattering resulting from the cellular morphological changes driven by the cell death. Tissues that undergo apoptosis scatter ultrasound due to nuclear condensation and fragmentation. The increased scattering of ultrasound from apoptotic cells results in considerable changes to spectral measurements, such as the mid-band fit. Therefore, larger changes in mid-band fit reflect greater tissue damage and greater levels of cell death [62,63]. The results indicate that the mid-band fit increased gradually from the untreated control condition (0.158 ± 0.031 dB of change) to the triple-combination therapy (9.396 ± 0.651 dB of change) (Figure 2A). The single treatments and double-combination treatments displayed intermediate levels of change in the mid-band fit, in a range of ~1–6 dB (Figure 2A). Compared to the untreated control, the single treatments of TXT (1.879 ± 0.352 dB change, *p* = 0.0224) and USMB (3.208 ± 0.826 dB of change, *p* < 0.0001) resulted in significantly larger changes in the mid-band fit (with the exception of the XRT condition, *p* = 0.4588). In turn, the double-combination treatments resulted in significantly larger changes in the mid-band fit compared to the single treatments (with the exception of the USMB vs. TXT + USMB comparison, *p* = 0.0742). There were no statistically significant differences between the double-combination therapies (refer to Table 3 for the *p*-values). Finally, the triple-combination therapy resulted in significantly greater changes in the mid-band fit than all the other treatments. Furthermore, the linear regression analysis displayed a positive linear correlation between the mid-band fit and the overall cell death (R^2^ = 0.9164, *p* < 0.0001), as well as a positive linear correlation between the mid-band fit and the apoptosis (R^2^ = 0.8530, *p* < 0.0001). These results indicate a correspondence between the histological and the spectral measurements of tissue microstructure and, once more, demonstrate an enhancement of radiotherapy in vivo with the triple-combination therapy.

### 3.3. Tumour-Growth Delay and Overall Survival

**Figure 3:**
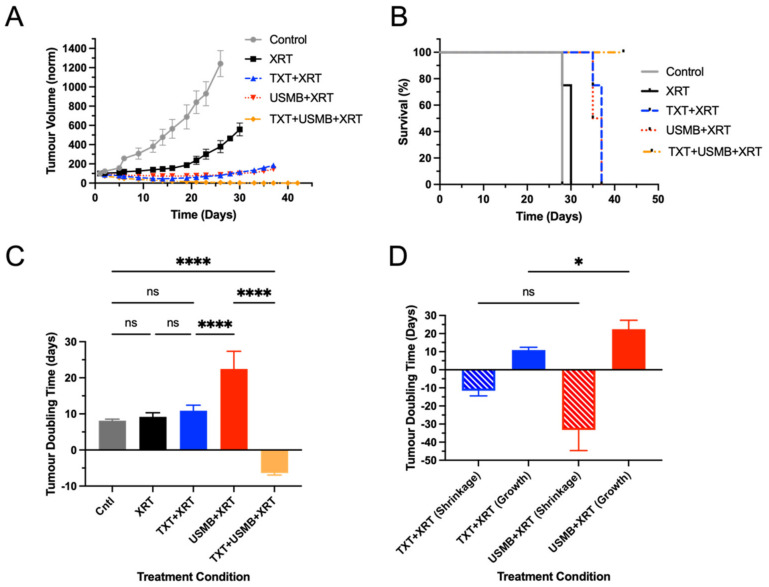
Tumor-growth curves and mouse-survival curves as functions of time. (**A**) Normalized tumor volumes (mm^3^) as a function of time (in days) for untreated control and four treatment conditions. (**B**) Percentage survival as a function of time (in days). (**C**) Tumor-doubling time (in days) for all five conditions. Doubling times were generated through Graphpad Prism by fitting growth curves to an exponential (Malthusian) model. (**D**) Tumor-doubling time (in days) for TXT + XRT and USMB + XRT groups separated into two parts: tumor-shrinkage phase (days 1–14) and tumor-growth phase (days 16–37). Four animals were used for each of the following conditions: untreated control (gray trendline), XRT (black trendline), TXT + XRT (blue trendline), USMB + XRT (red trendline), and TXT + USMB + XRT (orange trendline). Differences between tumor-volume means were found to be significant at an alpha level of 0.05 (see Table 4). Statistical significance: *p* > 0.05 (ns), 0.01 < *p* < 0.05 (*), *p* < 0.0001 (****).

A 42-day longitudinal study was conducted to assess the tumor growth and overall survival under five different treatment conditions, with four mice per condition. The conditions included in the study were untreated control, XRT, TXT + XRT, USMB + XRT, and TXT + USMB + XRT. The tumors from the untreated control group and the XRT group were similar for the first week, but the control tumors became significantly larger by day 6 (control doubling time = 8.12 days, Figure 3C). By day 2, the tumors from the untreated control were significantly larger than those from all the other groups and remained so over the course of the study. The mice exposed to XRT displayed tumor-growth inhibition during the first 16 days as a result of the relatively slow growth compared to the following days, by which point, disinhibition occurred, and the rate of tumor growth increased (XRT V_T_ = 9.19 days, Figure 3C). The tumor volumes of the mice treated with TXT + XRT and USMB + XRT were not significantly different from each other at virtually any of the time points. Both groups displayed an initial regression (shrinkage) in tumor size (days 1–14; TXT + XRT V_T_ = −11.56 days; USMB + XRT V_T_ = −33.20 days; not statistically different, *p* = 0.1290; Figure 3D) followed by a growth phase (days 15–37; TXT + XRT V_T_ = 10.90 days; USMB + XRT V_T_ = 22.45 days; *p* = 0.0259; Figure 3C,D). The doubling times for the controls, XRT, and TXT + XRT (growth phase) were not significantly different from each other, while the doubling times for the USMB + XRT (growth phase) were significantly greater than for all the other conditions (*p* < 0.0001). The tumors from the mice treated with the TXT + USMB + XRT exhibited a reduction in tumor volume, starting from day two, and at each subsequent time point measured (TXT + USMB + XRT V_T_ = −6.37 days). The mean tumor doubling time for the triple-combination group was significantly lower than for all the other groups (*p* < 0.0001). The tumor volumes from the triple-combination group were significantly lower than those in the control, XRT, USMB + XRT, and TXT + XRT groups from day two onward (Table 4). All the treatment groups became significantly different from each other (*p* < 0.05) from day two onward, with the exception of the control vs. XRT (*p* < 0.001 from day 6 onward), XRT vs. USMB + XRT (*p* < 0.001 from day 5 onward), and TXT + XRT vs. USMB + XRT (*p* < 0.05 on day 14 only). The mice within the triple-combination group were sacrificed at the 42-day study endpoint, at which point the tumors were undetectable visually and by means of palpation.

An ethical endpoint was reached at 28 days for the untreated control group as a result of rapid tumor growth (a > 10-fold increase in tumor size relative to day 1). The mice treated with the XRT survived longer than the mice in the untreated control group (untreated controls’ median survival—28 days; XRT median survival—30 days). The double-combination treatment groups survived past 30 days, with survival rates that were slightly higher in the TXT + XRT group than in the USMB + XRT group (TXT + XRT median survival—37 days; USMB + XRT median survival—36 days). Finally, all the mice from the TXT + USMB + XRT group survived the entire study course. Together, these results suggest that supplementing radiotherapy with both USMB and TXT leads to improved tumor-growth inhibition, a substantial reduction in tumor size (tumor ablation), and improvements in overall survival.

## 4. Discussion

Previous studies demonstrated the enhancement of radiotherapy (XRT) when it is combined XRT with either docetaxel (TXT) [48,49] or ultrasound microbubbles (USMB) [22,29,33,34], which we also observed in this study. We further demonstrated that combining TXT and USMB enhanced the therapeutic effects of XRT in a PC-3-prostate-carcinoma mouse model. In addition, we demonstrated that the TXT + USMB + XRT further increased tumor-cell death and apoptosis compared to single and double treatments. Furthermore, the triple-combination therapy led to substantial tumor shrinkage, resulting in tumor ablation within 5–6 weeks. These findings suggest that the triple-combination therapy of TXT + USMB + XRT may be an even more effective cancer therapy than standard treatments.

The mechanisms underlying USMB-induced chemo- and radioenhancement are not yet fully understood. However, the enhanced tumor responses attributed to USMB are associated with cavitating microbubbles and the resulting mechanical stress exerted on the endothelium of the tumor microvasculature. These stresses induce one or more of several bioeffects, such as changes in gene expression, increased endocytosis, membrane-pore formation, and cell death [30,64,65,66]. The enhanced therapeutic responses observed when combining USMB with chemotherapy are believed to be caused by the increased delivery of the drugs to the tumor environment through extravasation. The enhanced therapeutic response observed when combining USMB with radiotherapy is believed to involve the disruption of tumors’ vascular supply of nutrients and oxygen. Ample studies have demonstrated that radiotherapy encourages endothelial cell death and that USMB potentiates this cell-death response [23]. Indeed, studies using various tumor models showed that USMB increased endothelial cell death and decreased tumor vascularization when combined with XRT, leading to improved treatment responses. The primary mechanism explaining the synergistic effect of combining USMB with XRT is endothelial cell damage through the activation of the acid sphingomyelinase (ASMase)–ceramide pathway of cell death [22,28,35]. The enzyme ASMase, which is stored in lysosomes, converts sphingomyelin on the plasma membrane to ceramide, a secondary messenger involved in apoptosis signaling. As part of the cellular-stress response, extracellular Ca^2+^ flows into the cell to induce the fusion of lysosomes with the plasma membrane, encouraging the translocation of ASMase to the membrane, where it converts sphingomyelin to ceramide [67]. Several studies have shown the importance of the ceramide pathway in USMB-induced radioenhancement. Studies have demonstrated that the inhibition of ceramide production through sphingosine 1-phopshate (S1P) treatment or ASMase genetic knockout reduces the radioenhancement effects of USMB [22,23,27,28,30,35]. Moreover, the power-Doppler-ultrasound imaging of blood flow within xenograft tumors in mice showed reduced perfusion following treatment with USMB and XRT. However, vascular perfusion was unaffected when USMB + XRT therapy was given to ASMase knockout mice or mice pre-treated with S1P [28]. It is important to note that different tumor types/models were used across studies that employed relatively comparable study designs and treatment parameters; these studies employed single treatments of 2–8 Gy of ionizing radiation, Definity^TM^ microbubbles at concentrations of 1–3% *v*/*v* (based on animal-blood volume), and peak negative ultrasound pressures of ~570 kPa. Altogether, these findings suggest that the mechanism of USMB-induced radioenhancement is related to the perturbation of the endothelia of tumors via the ASMase–ceramide pathway [22,28,35,68].

As with all combination therapies, the study of the different permutations of treatment parameters is needed to determine an optimal treatment protocol, which would produce favorable tumor responses while minimizing side effects. Our study consisted of a three-treatment combination therapy, which required an investigation of its parameter permutations: the radiation dose and fractionation schedule, microbubble concentration, peak negative ultrasound pressure, and chemotherapeutic concentration. Some studies provide insight into optimal treatment parameters. For example, Czarnota et al. found that changing microbubble concentrations and radiation doses had lesser effects at high peak negative ultrasound pressures (>570 kPa) in a PC-3-prostate-carcinoma mouse model, whereas increasing the microbubble concentration and radiation dose at lower pressures (250 kPa) increased the tumor ceramide levels and cell death [33,35,68]. The time interval between USMB and XRT administration and the sequence regimen also warrant consideration. Klein et. al. found no difference between USMB-XRT and XRT-USMB treatment sequences while determining a 6-h treatment separation between XRT and USMB to produce the greatest treatment effects in a PC-3 prostate-carcinoma mouse model [69]. The exploration of the therapeutic benefits of a multi-USMB treatment sequence also warrants further investigation [32]. These factors should be taken into consideration for follow-up preclinical studies and translational clinical studies. While the treatment parameters require fine-tuning, the use of triple-combination therapy is promising in that it may allow clinicians to further decrease chemotherapy and radiation doses to minimize toxic side effects while achieving clinically relevant tumor responses [70].

## 5. Conclusions

Our study provides a proof-of-concept for the combination of USMB and chemotherapy (TXT) in the setting of radiotherapy to increase tumor responses in a prostate-carcinoma mouse model. The tumors treated with the triple-combination therapy displayed increased cell death and apoptosis compared to the single treatments and double-combination therapies. The cell-death results were corroborated by the ultrasound-radiofrequency imaging of the tumor microstructure. Finally, the triple-combination therapy induced tumor shrinkage and prolonged the survival rate beyond all those of all the other treatment groups. The tumors of these mice were undetectable through sight and palpation by the end of the study. Exploring the use of this triple-combination therapy for solid tumors warrants further attention to improve the efficacy and tolerability of current standard-of-care treatments.

## Figures and Tables

**Table 1:** Summary of cell death (H&E) statistics.

	Control	TXT	USMB	XRT	TXT + USMB	TXT + XRT	USMB + XRT	TXT + USMB + XRT
Control	-							
TXT	**	-						
USMB	*	ns	-					
XRT	*	ns	ns	-				
TXT + USMB	****	**	***	***	-			
TXT + XRT	****	****	****	****	ns	-		
USMB + XRT	****	***	****	****	ns	ns	-	
TXT + USMB + XRT	****	****	****	****	****	****	****	-

*p* > 0.05 (ns), 0.01 < *p* < 0.05 (*), 0.001 < *p* < 0.01 (**), 0.0001 < *p* < 0.001 (***), *p* < 0.0001 (****).

**Table 2:** Summary of apoptosis (TUNEL) statistics.

	Control	TXT	USMB	XRT	TXT + USMB	TXT + XRT	USMB + XRT	TXT + USMB + XRT
Control	-							
TXT	ns	-						
USMB	ns	ns	-					
XRT	ns	ns	ns	-				
TXT + USMB	****	**	**	*	-			
TXT + XRT	****	****	****	***	ns	-		
USMB + XRT	***	*	*	ns	ns	ns	-	
TXT + USMB + XRT	****	****	****	****	****	****	****	-

*p* > 0.05 (ns), 0.01 < *p* < 0.05 (*), 0.001 < *p* < 0.01 (**), 0.0001 < *p* < 0.001 (***), *p* < 0.0001 (****).

**Table 3:** Summary of statistics for changes in mid-band fit.

	Control	TXT	USMB	XRT	TXT + USMB	TXT + XRT	USMB + XRT	TXT + USMB + XRT
Control	-							
TXT	*	-						
USMB	****	ns	-					
XRT	ns	ns	**	-				
TXT + USMB	****	****	ns	****	-			
TXT + XRT	****	****	***	****	ns	-		
USMB + XRT	****	****	**	****	ns	ns	-	
TXT + USMB + XRT	****	****	****	****	****	****	****	-

*p* > 0.05 (ns), 0.01 < *p* < 0.05 (*), 0.001 < *p* < 0.01 (**), 0.0001 < *p* < 0.001 (***), *p* < 0.0001 (****).

**Table 4:** Summary of tumor-growth-curve statistics.

Day	Control vs. XRT	Control vs. TXT + XRT	Control vs. USMB + XRT	Control vs. TXT + USMB + XRT	XRT vs. TXT + XRT	XRT vs. USMB + XRT	XRT vs. TXT + USMB + XRT	TXT + XRT vs. USMB + XRT	TXT + XRT vs. TXT + USMB + XRT	USMB + XRT vs. TXT + USMB + XRT
2	*-*	0.001 (0.01)	0.003 (0.03)	<0.001 (0.002)	<0.001 (0.006)	*-*	<0.001 (<0.001)	*-*	0.002 (0.03)	<0.001 (0.01)
5	*-*	0.001 (0.01)	0.002 (0.02)	<0.001 (0.004)	<0.001 (0.002)	<0.001 (0.01)	<0.001 (<0.001)	*-*	0.003 (0.05)	<0.001 (0.007)
6	<0.001 (<0.001)	<0.001 (<0.001)	<0.001 (<0.001)	<0.001 (<0.001)	<0.001 (<0.001)	<0.001 (0.003)	<0.001 (<0.001)	*-*	0.003 (0.04)	<0.001 (0.005)
9	<0.001 (0.009)	<0.001 (0.002)	<0.001 (0.003)	<0.001 (0.001)	<0.001 (<0.001)	<0.001 (0.003)	<0.001 (<0.001)	*-*	*-*	<0.001 (0.003)
14	<0.001 (0.002)	<0.001 (<0.001)	<0.001 (<0.001)	<0.001 (<0.001)	<0.001 (<0.001)	<0.001 (<0.001)	<0.001 (<0.001)	0.002 (0.02)	<0.001 (0.01)	<0.001 (<0.001)
19	<0.001 (0.002)	<0.001 (<0.001)	<0.001 (<0.001)	<0.001 (<0.001)	<0.001 (<0.001)	<0.001 (<0.001)	<0.001 (<0.001)	*-*	<0.001 (0.001)	<0.001 (<0.001)
26	<0.001 (<0.001)	<0.001 (<0.001)	<0.001 (<0.001)	<0.001 (<0.001)	<0.001 (<0.001)	<0.001 (0.001)	<0.001 (<0.001)	*-*	<0.001 (<0.001)	<0.001 (<0.001)
30	N/A	N/A	N/A	N/A	<0.001 (<0.001)	<0.001 (<0.001)	<0.001 (<0.001)	*-*	<0.001 (<0.001)	<0.001 (<0.001)
37	N/A	N/A	N/A	N/A	N/A	N/A	N/A	*-*	<0.001 (<0.001)	<0.001 (<0.001)

The tabulation of the *p*-values for the differences in tumor volume between the groups at specified time-points across the 42-day study. Unbracketed *p*-values were derived from an unpaired Student’s *t*-test, and the bracketed *p*-values were derived from a post hoc Bonferonni–Dunn test for multiple comparisons. The cells denoted with “-” indicated no statistically significant differences with a Student’s *t*-test (*p* > 0.05), and the cells denoted with “N/A” indicate that at least one group was not present at the specified timepoint as a result of the study’s endpoint being reached (i.e., endpoint associated with tumor burden). At the onset of the tumor-growth study, the tumors from the untreated control mice were significantly smaller than those from the double- and triple-combination groups, but they became significantly larger within the first week post-treatment. The tumor volume in the XRT group was also significantly smaller than in the TXT + USMB + XRT group on day one, but it became significantly larger thereafter.

## Data Availability

The data presented in this study are available on request from the corresponding author.
